# Surface Molecularly Imprinted Polymer of Chitosan Grafted Poly(methyl methacrylate) for 5-Fluorouracil and Controlled Release

**DOI:** 10.1038/srep21409

**Published:** 2016-02-19

**Authors:** Xue-Fang Zheng, Qi Lian, Hua Yang, Xiuping Wang

**Affiliations:** 1 College of Chemical Engineering, Hebei Normal University of Science and Technology, Qinhuangdao 066600, China; 2 School of Chemistry and Chemical Engineering of Guangxi University, Nanning, 530004, China; 3 College of Life Science and Technology, Hebei Normal University of Science and Technology, Qinhuangdao 066600, China

## Abstract

The molecular surface imprinted graft copolymer of chitosan with methyl methacrylate (MIP-CS-g-PMMA) were prepared by free radical polymerization with 5-fluorouracil (5-FU) as the template molecule using initiator of ammonium persulfate as adsorption system. MIPs were characterized by FTIR, X-ray diffraction, thermo-gravimetric analysis, ^1^H NMR and SEM. The mechanism of graft copolymerization and factors affected graft reaction were studied in details, and the optimum reaction conditions (to the highest %G and %E as the standard) were obtained at [MMA] 1.2 mol/L, [Chitosan] 16.67 mol/L, [initiator] 0.0062 mol/L, temperature 60 °C and reaction time 7 h. MIPs exhibited high recognition selectivity and excellent combining affinity to template molecular. The *in vitro* release of the 5-FU was highly pH-dependent and time delayed. The release behavior showed that the drugs did not release in simulated gastric fluid (pH = 1.0), and the drug release was small in the simulated small intestinal fluid (pH = 6.8), and drug abrupt release will be produced in the simulated colon fluid (pH = 7.4), indicating excellent colon-specific drug delivery behavior.

In recent years, a growing attention has been focused on the field of controlled drug release delivery to the polymer matrix oral colon-specific drug delivery system (OCDDS), which has been developed positively. An ideal colon-specific drug delivery system should prevent from releasing in the stomach and small intestine, and produce an abrupt onset of drug release upon the entry into the colon, which aims at not only increasing the local concentration of the drug in the site of the lesion, but also reducing systemic side effects and improving the drug efficacy and safet^1^,^2^,^3^. OCDDS has important prospects in the treatment of colon cancer and colitis and other diseases, and in terms of oral administration of therapeutic circadian rhythm disorders drugs, peptides and proteins^4^,^5^. Building and developing efficient OCDDS new formulation is an important research topic in the cross field of chemistry and medicine by the use of physical and chemical properties of the polymer materials and drugs through molecular design ideas.

Molecular imprinting is a now well-established way as one of the most promising technologies for the preparation of intelligent polymer materials with specific recognition capacities for template molecules^6^,^7^,^8^,^9^. In recent years, molecularly imprinted polymers(MIPs) have attracted much attention because of their outstanding advantages, such as predetermined recognition ability, stability, relatively ease and low cost of preparation, and potential application to a wide range of target molecules^10^,^11^,^12^. In the most common preparation process, monomers form a complex with a template molecule through covalent or non-covalent inter-actions and are then joined by using a cross-linking agent. After removing of the imprinting molecules by chemical reaction or extraction, the binding sites (binding sites) are exposed which are complementary to the template molecule in size, shape, and position of the functional groups. If the template molecule is a medicinal substance, the imprinted caves are visible as a drug reservoir because there is a strong binding force to the molecules, resulting in effectively delaying the release process of drug molecules. Furthermore, according to the external environment (such as pH, salt concentration, and temperature), environmentally sensitive type of controlled release drug delivery system can be produced with the methods of changing the binding capacity of the drug molecule in the imprinted caves to control drug release process. Therefore, molecular imprinting technique is also beginning to be used for controlled release drug delivery system, in order to obtain high performance controlled release system^13^,^14^,^15^.

Chitosan is produced by the deacetylation of chitin, which is known to be the most abundant natural amino-polysaccharide presenting non-toxicity, bio-degradability and bio-compatibility. The presence of multiple functional groups such as amino and hydroxyl groups on its polysaccharide chain provides the flexibility for structural modifications and for preparing molecularly imprinted polymers. It is, therefore, interesting not only as an abundant resource but also as a novel type of functional materials. The applications have been enormously expanded in various fields including biotechnology, water-treatment, membranes, cosmetics, food industry, and medicine^16^,^17^,^18^,^19^,^20^,^21^. However, chitosan is poor in water and in common organic solvents, which restricts the application in many fields. Chitosan, due to the presence of -OH and -NH_2_ on the backbone, could be introduced by different groups by chemical modification. Derivatives prepared, because of the introduction of different substituents. The modification of chitosan, on the one hand can improve the solubility of chitosan, on the other hand can introduce desired properties and enlarge the application fields of chitosan^22^. Therefore, the chemical modification of chitosan is one of the most active areas of research. However, few works with the combination of chitosan in molecular imprinting are reported^23^. Nonetheless, few studies have been reported on the preparation of MIPs with chitosan grafted poly (methyl methacrylate), and no study has been reported about the pH-sensitive properties of molecular recognition. Hence, MIPs should be suitable as an ideal polymer material for wider applications of chitosan.

5-fluorouracil (5-FU) is the best chemotherapy drug of colorectal cancer with the advantages of a wide spectrum of anti-cancer and high efficiency, but there are the following disadvantages: fast metabolism, short half-life (10 ~ 20 min), low bioavailability, large cytotoxicity, and poor selectivity for tumor cells. Oral formulations would cause serious gastrointestinal reactions and side effects such as bone marrow toxicity. In order to overcome above shortcomings of 5-FU, and improve the chemotherapy efficiency of colorectal cancer, many groups^24^,^25^ have focused on the development of oral colon-specific 5-FU delivery system which can avoid releasing of the oral 5-FU in the stomach and in the front of the small intestine, and can transfer the drug to release at the site of colon to increase the local concentration of the drug in the lesion.

Based on the persulfate initiating the surface graft polymerization, we design and prepare a new oral colon-specific 5-FU delivery system with molecular surface imprinted technique with CS-PMMA as matrix microspheres and with the 5-FU as template molecule, which is the combination of the pH-development and time-delayed release mechanisms. The oral colon-specific 5-FU delivery system will be hoped to apply for treatment of colon and colorectal cancer with high efficacy and safety.

## Results and Discussions

### Mechanism of 5-FU imprinted microspheres MIP-CS-g-PMMA

**Figure 1:**
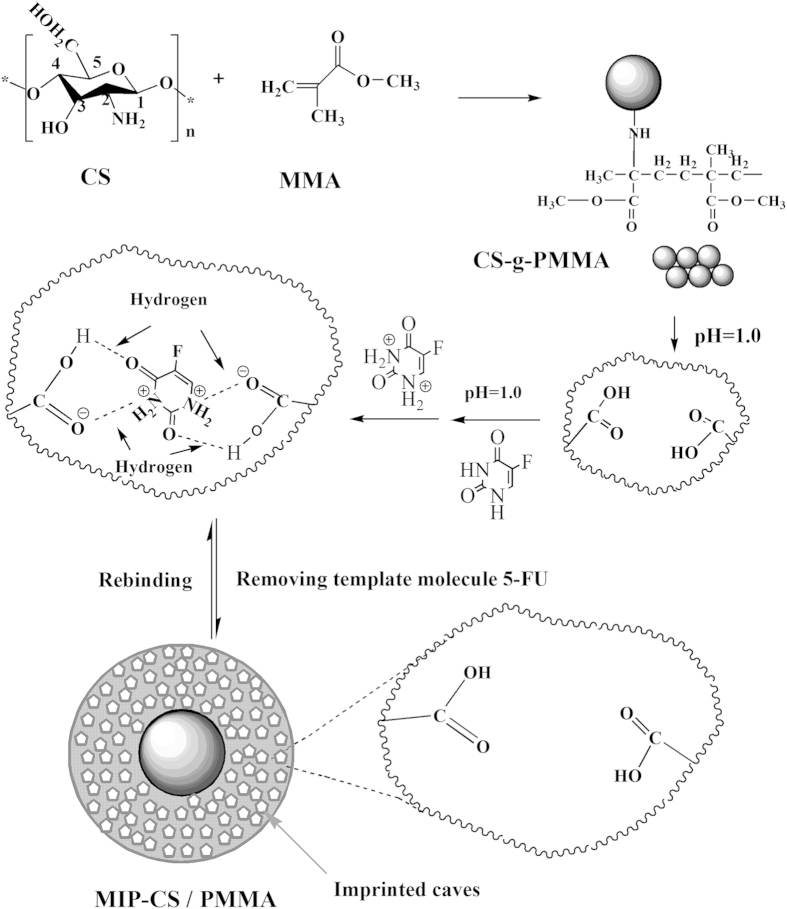
Schematic expression of chemical reaction process to prepare 5-FU imprinted microspheres MIP-CS-g-PMMA.

The graft copolymerization of chitosan was prepared with the initiator of ammonium persulfate. In acidic solution, the ester group of copolymerization was hydrolyzed to group of –COOH, and oxygen atom of carboxyl group had strong electrical negative with a unit of negative charge. On the other hand, the template molecule of 5-fluorouracil contained two amide groups which were easily protonated, resulting that 5-fluorouracil was carried a positive charge, which was like a large proton and could form hydrogen bonds with a group of carboxyl, but there were almost completely ionized with each other. Therefore, they were also called ionic hydrogen bonds, which were stronger than common hydrogen bonds^26^. Therefore, the molecules of 5-FU were embedded in the cross-linked network, and the molecular imprinted polymer of 5-FU was prepared successfully. After removing of the imprinting molecules by chemical reaction or extraction, binding sites are exposed which are complementary to the template molecule of 5-FU in size, shape, and position of the functional groups, and consequently allow their selective uptake (Fig. 1).

### Characterization of the MIP-CS-g-PMMA

#### FT IR analysis

**Figure 2:**
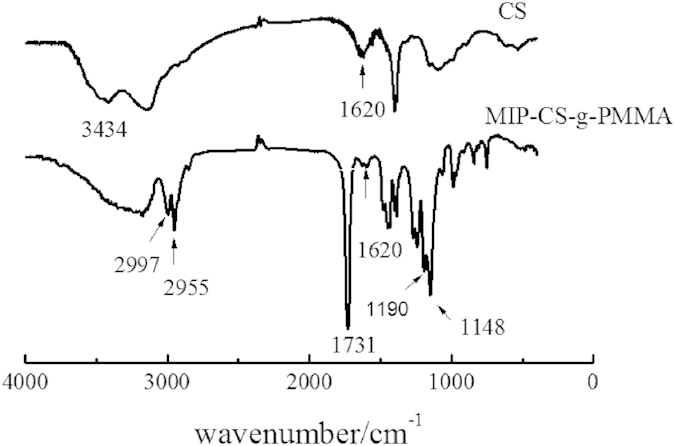
FTIR spectra of chitosan and MIP-CS-g-PMMA.

Figure 2 showed the IR spectra of chitosan and MIP-CS-g-PMMA. The strong peaks at 3434 cm^−1^ was attributed to the stretching vibration of O-H. The sharp absorption peaks of MIP-CS-g-PMMA at 1725, 2955 and 2997 cm^−1^ were assigned to the carbonyl stretching and symmetrical and asymmetrical stretching of the methyl group, respectively^27^,^28^. In the spectra of MIPs, the peaks at 1620 cm^−1^ (due to N-H plane deformation vibration absorption) became weak, and the peaks at 1148 cm^−1^ (assigned to C-N stretching) became significantly enhanced. These provided evidences that the PMMA had grafted onto the chitosan. The bands in the region of 3000 cm^−1^–3500 cm^−1^, relating to the intensity of the hydrogen bonds, were broader in the spectra of copolymerization than chitosan, indicating that there were more hydrogen bonds in the MIPs and this could generate advanced performance of molecular imprinting.

#### XRD analysis

**Figure 3:**
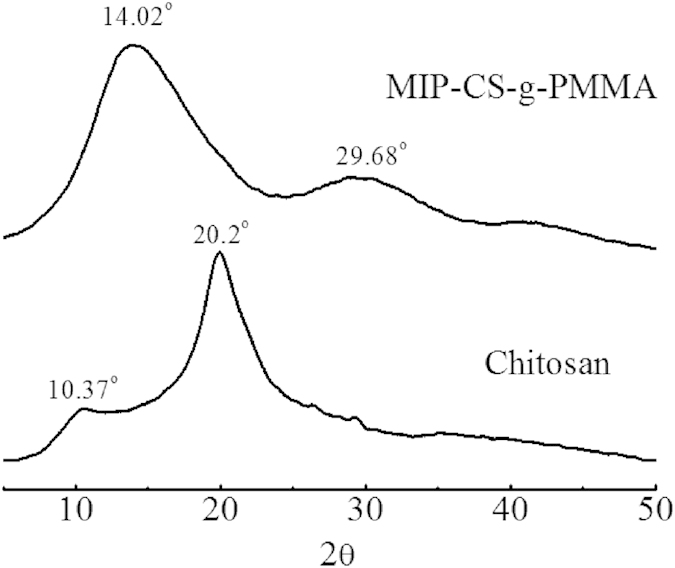
X-ray diffractograms of chitosan and MIP-CS-g-PMMA.

Figure 3 showed the spectra of x-ray diffraction (XRD) of MIPs and chitosan^29^. The crystalline peaks (2θ) at 10.3° and 20.2° were attributed to chitosan, which are typical fingerprint for chitosan and very similar to the work of Wan, Creber, Preppley, and Bui^30^. From previous report^31^, the small peak at approximately 10° (2θ) was attributed to the anhydrous crystal of chitosan whereas the diffraction peak at around 21–22° (2θ) was observed in chitosan prepared from dissolving chitosan in acetic acid solution^32^,^33^,^34^. While after PMMA grafting, it acquires crystalline areas around 14.02° (2θ) and 30° (2θ) (Fig. 3) in the copolymerization. This was because numerous of hydrogen bonds formed to destruct the original crystalline structure of the chitosan during the synthesis, which indicated that the PMMA had grafted onto chitosan successfully.

#### TGA analysis

**Figure 4:**
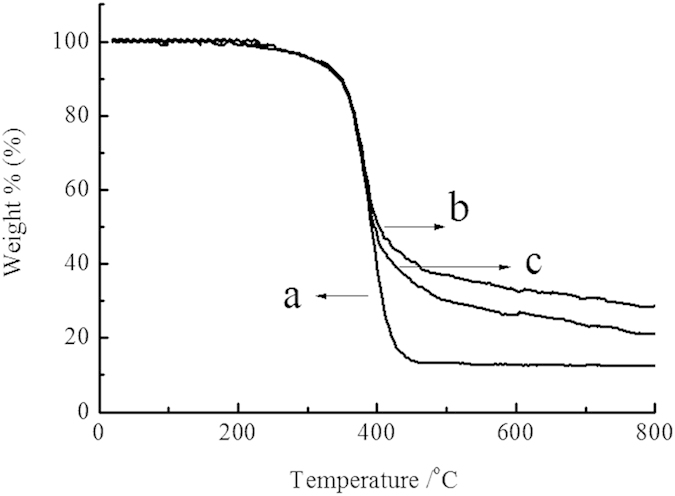
TGA for (**a**) original chitosan, (**b**) CS-g-PMMA (%E = 47) and (**c**) CS-g-PMMA (%E = 21).

Figure 4 showed the TGA curves of the original chitosan and CS-g-PMMA with different efficiency of grafting. Curves of DTG derived from the derivation of TGA indicated that thermal decomposition temperature of curve (a) was around 430 °C, while curve b and c was approximately 410 °C. There are a little difference between (a), (b) and (c). However, as shown in Fig. 3, the thermal residual of the three curve was significantly different: (a) 10%, (b) 27% and (c) 21%, respectively. The reason may be that the process of TGA in N_2_ atmosphere was accompanied with the carbonization of PMMA. In the range of 0–410 °C, thermal decomposition all occurred in the three samples, but in the range of 410–800 °C, carbonization did not occur in (a), but happened in the graft copolymer (b) and (c) due to the presence of PMMA. The more is the %E, the higher is the residual rate^35^. This further confirmed the success of grafting reaction.

#### ^1^H NMR analysis

**Figure 5:**
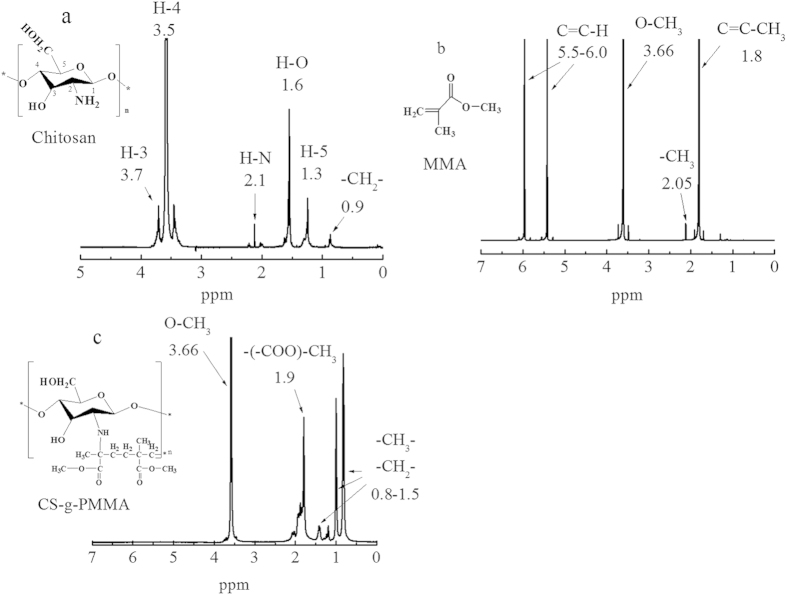
^1^H NMR spectra of (**a**) original chitosan, (**b**) pure PMMA and (**c**) copolymerization CS-g-PMMA (%E = 47) dissolved in D_2_O.

Figure 5 showed the structure of monomer of chitosan (a), MMA(b) and graft copolymerization CS-g-PMMA(c) with %E = 47. In Fig. 5(a), the peak at 0.9 ppm was attributed to the -CH_2_- of chitosan, and the peaks at 2.1 ppm was ascribed to the H-N of chitosan. The peak at 1.6 ppm was belonged to the –OH of chitosan. The H-3, H-4 and H-5 protons were found around 3.7 ppm, 3.5 ppm and 1.3 ppm, respectively. In spectra (b), the peaks at 1.80, 2.05 and 3.66 ppm were assigned to the C=C-H, C–CH_3_ and O-CH_3_ of MMA, respectively. And the peaks at 5.5 and 6.0 ppm to –C=C-H. By comparison, new peaks between 0.8 to 1.5 ppm in Fig. 4(c) were ascribed respectively to CH_3_, -CH_2_-, and the peak at 1.9 ppm was belonged to –COO-CH_3_ of the copolymerization CS-g-PMMA. And the peak at 2.1 ppm was obviously weakened, which demonstrated that amino was participated into the chemical reactions and generated the polymer CS-g-PMMA. Moreover, the peaks at 5.5 and 6.0 ppm had been disappeared in Fig. 4(c) which illustrated that the group of C=C of MMA had been consumed completely. Therefore, these once again confirmed that the MMA had grafted to chitosan successfully, because that there contained large amounts of methyl and methylene in MMA.

#### SEM analysis

**Figure 6:**
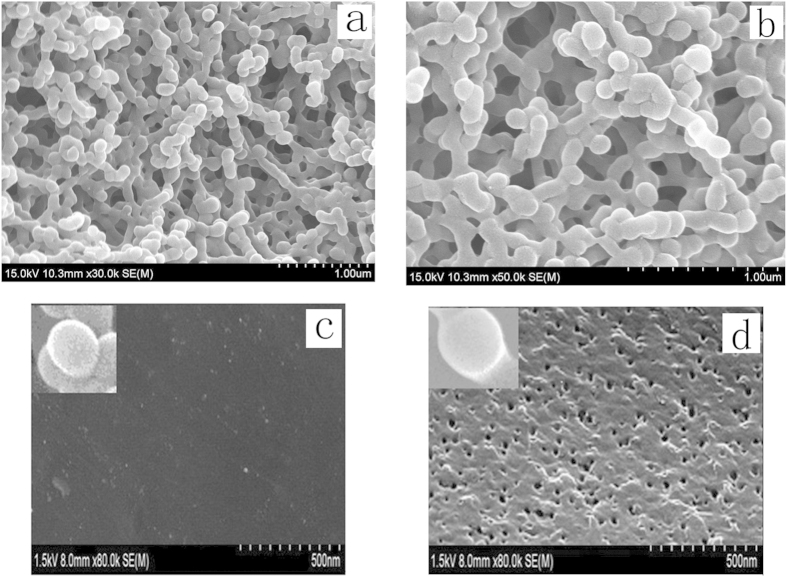
The SEM images of NIP-CS-g-PMMA (**a**), MIP- CS-g-PMMA (**b**), surface morphology of the NIP-CS-g-PMMA (**c**), and surface morphology of the MIP- CS-g-PMMA (**d**).

Figure 6 demonstrated the SEM image (a) NIP-CS-g-PMMA, (b) of MIP-CS-g-PMMA, the surface morphology of the NIP-CS-g-PMMA (c), and surface morphology of the MIP- CS-g-PMMA (d). As shown from image (a) and (b), the graft copolymerization was a unimodal size distribution within 150–200 nm, indicating that the nanoparticles were a relatively homogeneous dispersion. However, the diameter of MIPs was about 130 nm, which was bigger than that of NIPs with the diameter of 100 nm. The phenomenon was because the molecules of 5-FU were embedded in the network of MIP-CS-g-PMMA. From the Fig. 6d, the surface of MIPs was more rough than that of NIPs (Fig. 6c) after modified by template molecule of 5-FU, a large quantity of well-distributed cavities were left and observed after removing the template molecule of 5-FU. The porous structure on the surface of MIPs facilitated the mass transfer rate of releasing and the recognition ability of template molecule.

### Effect of the reaction parameters on the graft copolymerization

**Figure 7:**
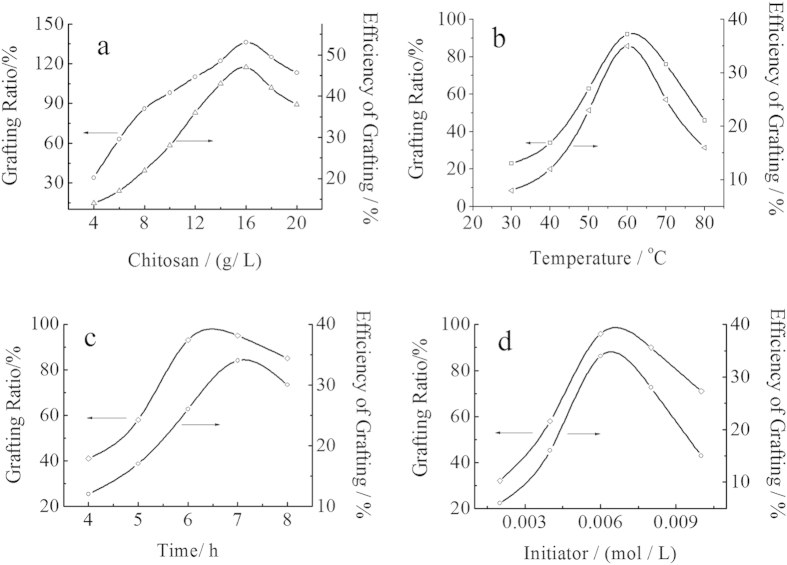
Effect of reaction conditions on the grafting rate and grafting efficiency. (**a**) Effect of chitosan concentration. Reaction conditions: [MMA] 1.2 mol/L, [initiator] 0.0062 mol/L, temperature 60 °C, reaction time 7 h. (**b**) Effect of the reaction temperature. Reaction conditions: [MMA] 1.2 mol/L, [initiator] 0.0062 mol/L, [Chitosan] 16.67 mol/L, reaction time 7 h. (**c**) Effect of reaction time. Reaction conditions: [MMA] 1.2 mol/L, [initiator] 0.0062 mol/L, [Chitosan] 16.67 mol/L, temperature 60 °C. (**d**) Effect of the initiator concentration. Reaction conditions: [MMA] 1.2 mol/L, [Chitosan] 16.67 mol/L, temperature 60 °C, reaction time 7 h.

Figure 7 showed the effect of the chitosan concentration, reaction temperature, reaction time and initiator concentration on the grafting rate and grafting efficiency under the indicated reaction conditions. As can be seen from Fig. 7(a), when the concentration of chitosan increased, the %G and %E both increased immediately. This phenomenon can be explained from the graft mechanism. With increasing the concentration of chitosan, the total number of group -NH_2_ of chitosan increased, resulting in more activated sites of chitosan, which meant that there were more opportunities for MMA to attack the radicals of chitosan. Therefore, %G and %E both increased with increasing the concentration of chitosan. However, when the concentration of chitosan was too large, the system became so thick that the radicals of chitosan could not move freely, resulting in the decrease of %G and %E of the products. So, in the further experiment, the concentration of chitosan was 16.67 g/L.

The influence of temperature on graft parameters was studied, varying the reaction temperature from 30–80 °C with other parameters being fixed. As known in Fig. 7(b), in the initial stage of the reaction, %G and %E both increased with increasing the temperature from 30–60 °C, and then decreased. This behavior could be interpreted that the activity of the initiator increased with the increase of temperature, which was helpful to improve the activity of both graft copolymerization and homopolymerization. However, when the temperature exceeded 60 °C, the initiator ability of free radicals reduced with the decrease of the stability of the active center, resulting in decreasing both %G and %E. On the other hand, with increasing the temperature, the mobility of macro radicals would be improved, and more prone to the chain termination at higher temperature^36^. Thus, the polymerization activity began to decline, and %G and %E of the graft copolymer were decreased at the elevated temperature.

The influence of reaction time on the graft parameters was investigated by changing the reaction time from 4 to 8 hours, keeping other parameters fixed. As can be seen in Fig. 7(c), the %G and %E both increased with the increase of reaction time, and then decreased. The grafting ratio was maximum in 7 hour of the reaction time. But when the time was more than 7 hour, %G and %E decreased with the increase of the side effects in the copolymerization.

Figure 7(d) showed the effect of the initiator concentration on the grafting efficiency. %G and %E both increased with the increase of the initiator concentration at the first stage of the reaction, and then decreased. The maximum of %G was achieved at around 0.0062 mol/L of the concentration of the initiator. When the concentration of initiator was 0.002 mol/L, the grafting efficiency of copolymerization was lower (about 6.2%). With the initiator concentration increased, the active center formed by the initiator and chitosan increased, resulting in the increase of %G and %E. However, when the concentration was larger than 0.006 mol/l, excessive free radicals would trigger plurality of coupling, so that the homopolymerization of MMA played a dominant role in the reaction.

### Swelling rate

**Figure 8:**
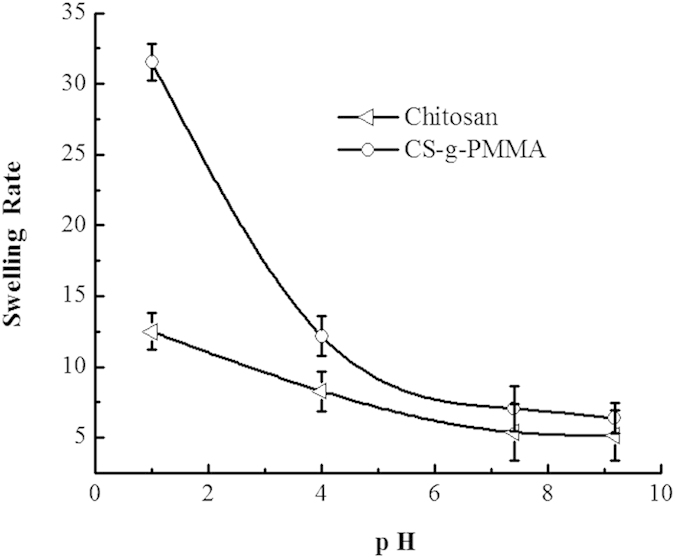
The swelling rate of polymer CS-g-PMMA and pure chitosan in different pH solution at 37 ± 0.5 °C.

As can be seen from Fig. 8, the swelling rate (SR) of polymer CS-g-PMMA and pure chitosan (CS) in different pH buffer solution was decreased with the increase pH value, and the SR of polymer CS-g-PMMA was smaller than that of pure CS. This phenomenon could be explained that, at pH 1.2, the swelling rate of pure chitosan was much larger than under other conditions (pH 4.0, 6.8, 7.4), because the gap of chitosan grids was increased by the protonated -NH_2_ of chitosan. As for polymer CS-g-PMMA, the swelling rate was much smaller than that of pure chitosan, which mainly because that -NH_2_ of chitosan was consumed to the polymer CS-g-PMMA with MMA, resulting that the protonated capability was greatly weakened, especially at pH 1.2.

### Adsorption and recognition properties of MIP-CS-g-PMMA

#### Binding characteristic of MIP-CS-g-PMMA

**Figure 9:**
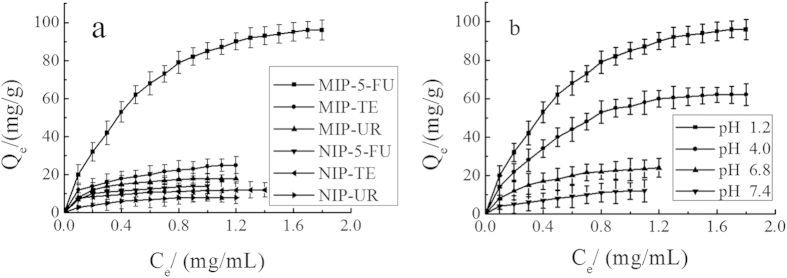
Effect of drug loading. (**a**) Effect of different drug molecules (5-FU, TE, UR) at room temperature in pH 1.2 hydrochloric acid solution of MIP and NIP. (**b**) Effect of different pH values (1.2, 4.0, 6.8, 7.4) on the drug loading for 5-FU of MIP at room temperature. (mean ± SD, n = 3).

**Figure 10:**
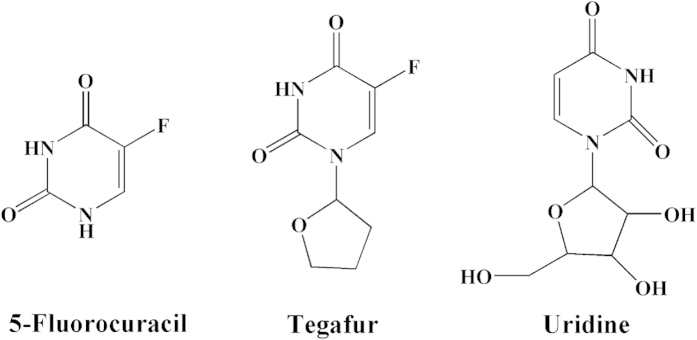
Molecular structures of 5-fluorouracil(5-FU), Tegafur(TE) and uridine(UR).

The adsorption and the recognition capacity are significant factors to evaluate the special binding of MIPs. Figure 9(a) showed the effect of drug molecules (5-FU, TE, and UR) on the drug loading of MIP and NIP. As can be seen from Fig. 9(a), the adsorption capacity of MIP-CS-g-PMMA microspheres for 5-FU, TE and UR was 96 mg/g, 25 mg/g, and 18 mg/g, respectively. The adsorption capacity of 5-FU was much higher than TE and UR. It is due to that there were a large number of cavities of 5-FU molecule distributed in the polymer MIPs which were highly matched with 5-FU not only in size but also in binding sites. Therefore, MIPs imprinted microspheres have the properties of both specific identification selectivity and high degree of affinity for binding. The binding capacity of TE and UR was very low, which can be explained that the molecular size of TE and UR was bigger than 5-FU (Fig. 10) and did not match with the imprinted holes, and hardly enter these imprinted cavities among the polymer of MIPs.

As for NIPs, the binding capacity for 5-FU, TE and UR was (about 14 mg/g, 11 mg/g, 8 mg/g) all low, which was because that any caves imprinted molecules were not formed and there were no binding sites to match for any molecule.

#### Effect of pH value on binding capacity (Drug loading capacity)

Figure 9(b) displayed the effect of different pH values (1.2, 4.0, 6.8, 7.4) on drug loading for 5-FU of MIP. The binding capacity for 5-FU (in pH 1.2, 4.0, 6.8, 7.4) was 96 mg/g, 62 mg/g, 24 mg/g, and 12 mg/g, respectively. As can be seen from Fig. 9(b), the binding capacity increased rapidly with the decreasing of the pH values, and the binding capacity reached the maximum (about 96 mg/g) in the buffer solution of pH 1.0 value. This phenomenon can be explained that the electrostatic force was very strong between blot holes and the template molecule 5-FU, because of the highly protonated amine 5-FU in acidic medium, resulting in high binding capacity to 5-FU of MIPs. However, in neutral or alkaline buffer solution, the electrostatic force was very weak with the low degree of protonation of the amine 5-FU, so that the binding capacity was decreased with the increase of the pH value.

The selectivity test of MIPs was carried out under equilibrium binding conditions using 5-fluorouracil(5-FU), Tegafur(TE) and uridine(UR) as substrates. The ratio of adsorption amounts obtained between the MIPs and NIPs was also compared. Here, the value of the ratio α(imprint factor) for each substrate is defined in the following equation^37^:


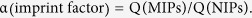


Table 1 showed the adsorption amounts of two substrates on the MIMs and NIMs. The imprint factor (α) of 5-FU, TE and UR was 6.86, 2.27 and 2.25, respectively. The result indicated that the MIPs exhibited higher selectivity for 5-FU on the absorption. This is because the imprinting process created binding sites with shape and functional group complementary to the template molecule, resulting in the MIPs having high selectivity for the template molecule, while TE and UR had no complementary shape and functional group matched with the imprinting cavities, resulting in the low binding capacity.

### *In vitro* controlled release behavior of drug-loaded microspheres

#### Release behavior in solutions with different pH values

**Figure 11:**
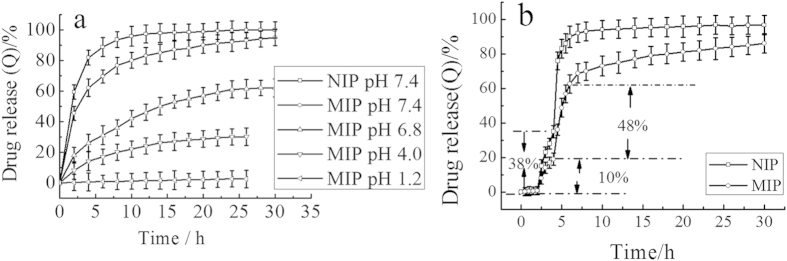
Effect of cumulated release of 5-FU at 37 °C. (**a**) Effect of different pH values (1.2, 4.0, 6.8, 7.4) on the cumulated release of 5-FU. (**b**) *In vitro* release profiles of drug carried microspheres in simulated gastrointestinal fluid at 37 °C. (mean ± SD, n = 3).

100 mg eluted polymers were immersed in hydrochloric acid buffer solution of 5-FU (containing 18 mg 5-FU in 10 ml, pH 1.2). The contents of 5-FU adsorbed by MIPs and NIPs were 96 mg/g and 14 mg/g, respectively. After separated by filtration and drying under vacuum, the polymers were added to a buffer solution with different pH, and the release kinetics of 5-FU from MIP and NIP were analyzed by the UV-vis detector. As can be seen from Fig. 11(a), the drug loaded microspheres of MIPs were not substantially released in hydrochloric acid buffer solution with pH 1.2. However, the cumulative release of 5-FU was increased with the increase of pH value of the buffer solution. And in pH 7.4 medium, the MIPs drug release of 5-FU was 48% in the first two hours, and the total drug release was reached 95% in 30 hours. But in the buffer solution (pH 6.8, 4.0 and 1.2), the cumulative drug release of MIPs were 62%, 30%, and 2.5%, respectively.

The swelling rate (at pH 1.2) was larger than under other conditions (pH 4.0, 6.8, 7.4), but the drug release rate (at pH 1.2) was the slowest. This phenomenon can be explained that, at pH 1.2, 5-FU with highly protonated to amine was tightly bound by the electrostatic forces in MIPs, resulting in the low drug release rate (*t*_*2.5%*_ = 30 h). The degree of amine protonated of 5-FU was diminished with increasing the pH value, so that the electrostatic force was weakened between microspheres imprinted cavies and template molecule of 5-FU, resulting in the increase of drug release rate. In the medium of pH 7.4, the degree of protonation of amine of 5-FU was greatly reduced, a large number of 5-FU molecules was released from MIPs in a short time (*t*_*25%*_ = 2 h, *t*_*90%*_ = 30 h). The MIP-CS-g-PMMA exhibited pH sensitivity with the drug release in different medium.

For NIPs, as can be also seen from Fig. 11(a), 61% of 5-FU was released from NIPs in the initial 2 hours, which was much larger than that of MIPs (*t*_*48%*_ = 2 h). The reason for this was that the absorption of 5-FU by NIPs was of physical adsorption to the surface of the polymers which was released in a short time because of the weak interaction between template molecule and polymers. However, for MIPs, 5-FU of rapidly from MIPs was adsorbed on the surface of the polymers, which may not get access to the long time diffusion of the aqueous medium. Then, in the following stage, the release of 5-FU slowed down with the controlled release behavior. This was due to that 5-FU was bundled in the deeper binding sites. Therefore, the release of 5-FU slowed down in the following time period. The MIP-CS-g-PMMA exhibited also time delay of release manner.

#### Release behavior in stimulated GI fluids

By using 0.1 M HCl solution (pH 1.2), PBS with pH 6.8 and another PBS with pH 7.4 as simulated gastric, small intestine and colonic fluids, respectively, and by referring to the drug actual running time in the gastrointestinal fluids, the cumulative drug release rate of drug-loaded microspheres graft NIP-CS-g-PMMA and MIP-CS-g-PMMA were measured under stirred by UV-vis for a certain period of time in different pH medium which was clearly showed in Fig. 11(b).

As can be seen from Fig. 11(b), the drug-loaded MIPs and NIPs were both hardly released with soaking in simulated gastric fluid for 2 hours. Soaking and stirring in the simulated small intestine of 2–4 hours, the drug release of MIPs and NIPs were 20% and 38%, respectively. The cumulative release ratio of the latter was almost twice as much as that of the former, because specific imprinted cavities on MIPs retaining a significant percentage of drug release. Once the polymers of MIPs was transferred to the simulated colonic fluids (pH 7.4), the drug release ratio increased significantly (*t*_*48%*_ = 4–6 h), exhibiting good feature of colon specific release, which was much lower than that of NIPs (*t*_*57%*_ = 4–6 h). This phenomenon may be interpreted that the drug can substantially retained on the surface of the microspheres of the MIPs in the small intestine, so that a large number of drugs burst release was occurred at the end of the small intestine and the front of the colon, to achieve a more efficient colon release, which was the OCDDS objectives pursued by many researchers^36^. The cause of the difference lied: drug-loaded microspheres MIPs imprinted hole to 5-FU molecule binding force made release behavior with time-delay.

## Methods

### Materials

Chitosan (CS, deacetylation degree of 90%) was purchased from Yuhuan Factory, China. Ammonium persulfate and Methyl methacrylate (MMA) of analytical grade with purity of 98% were used as the initiators and were applied by the Company of Tianjin Chemical Reagent. 5-fluorouracil (5-FU), Chicken protease and Pepsin with the purity of 95% were provided by Beijing Soledad Bao Technology Co. Ltd., China. All commercially solvents and reagents were used without further purification. All other chemicals were of analytical grade.

#### Synthesis of Molecular imprinting polymer Chitosan-g-poly (methylmethacrylate) (MIP-CS-g-PMMA)

A desired quantity of chitosan and 3 wt% acetic acid aqueous (with the total volume of the aqueous solution 60 ml) were filled in the 250 mL three necked flask. After the chitosan was dissolved in the acetic acid aqueous solution completely, the temperature of the system was controlled at a desired temperature. And Ammonium persulfate (0.1 wt%, 8.5 ml) was added into the system and the mixture was stirred for 30 min. After 5 min, a desired volume of methyl methymethacrylate (MMA, used for the monomer) were added to the graft copolymerization reaction. After 30 min, a desired quantity of 5-fluorouracil (used for template molecule) was also added into the solution.

At the time of generating white nanoparticles, the system was continuously stirred for a desired time, and then the mixture was centrifuged to obtain the solid. The solid product was washed by distilled water and acetone for repeatedly to pH = 7, then washed by anhydrous alcohol to remove the template molecular, salts and small molecular substances, and centrifuged for 3 times. The centrifuge was dried at 60 °C to a constant weight. Therefore, the craft copolymerization molecular imprinting polymer chitosan-g-poly (methylmethacrylate) (MIP-CS-g-PMMA) was obtained.

For a comparison, non-molecularly imprinted polymers (NIP- CS-g-PMMA) was prepared in the absence of template molecule (5-fluorouracil) during the polymerization process and treated in the identical manner.

All the samples were dried before used for characterization. Grafting rate (%G), which represents the backbone of the graft polymer in the amount of substrate (chitosan), and efficiency of grafting (%E), which indicates the initial conversion of MMA to the grafted PMMA, were calculated from the increase in weight of the chitosan after graft copolymerization in the following formula^38^:


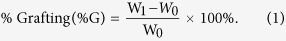



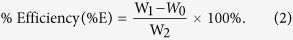


where W_1_, W_0_ and W_2_ denote the weight of the grafted chitosan, the weight of original chitosan and the weight of the monitor used, respectively.

### Swelling behaviour

To evaluate the water sorption resistance of the pure chitosan and polymer CS-g-PMMA, dry samples were weighed (*W*_*i*_) and then immersed in distilled water at 30 °C with shaking (100 rmp) for up to 24 h. Swollen samples were removed from water periodically, blotted dry, and weighed (*W*_*f*_) to track sporting kinetics. The swelling rate (*SR*) was determined as described by others^39^.


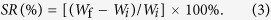


### Characterization

The structure of samples were characterized by Fourier transform infrared spectroscopy (FT-IR, IFS 66/S), Thermo-gravimetric analysis (TGA, ZRT-A/B), proton nuclear resonance (^1^H-NMR, INOVA-400), X-ray powder diffraction (XRD, D/max-3C), and Scanning electron microscopy analysis (SEM- Kyky-2800).

### Adsorption and selectivity experiments

100 mg MIPs or NIPs were soaked in the 5-fluorouracil solution with the concentration range from 0.2 mg/ml to 1.8 mg/ml for 12 hours to the adsorption equilibrium at room temperature, and then 0.4 ml supernatant was withdraw and determined with UV-vis(λmax = 265 nm) after dilution to a certain volume. The equilibrium binding capacity of 5-FU (*Q*e) was calculated according to the formula (4) as follows.


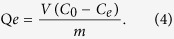


where *Q*_*e*_ denotes the equilibrium binding capacity of 5-FU (mg/g), *C*_0_ is the initial concentration solution (mg/L); *C*_e_ is the substrate concentration of the solution after adsorption equilibrium (mg/L); *V* and *m* signify the volume of the adsorbed solution (ml) and the mass of the MIPs or NIPs (g).

Molecular recognition of MIPs was adopted in the following manner that 100 mg MIP or NIPs were respectively immersed in the solution of 5-FU, tegafur (TE) and uridine (UR), and the concentration of the solution was monitored by UV-vis at the maximum absorption wavelength (λ_5-FU_ = 265 nm, λ_TE_ = 271 nm, λ_UR_ = 254 nm).

### *In vitro* controlled release of 5-fluorouracil

100 mg of MIPs or NIPs of CS-g-PMMA were immersed in 20 ml of 10 mg 5-fluorouracil in sodium hydroxide solution for 12 h at room temperature, and then were separated by filtration and dried under vacuum. MIPs or NIPs loading with 5-fluorouracil were suspended in 40 ml 0.1 mol/ l phosphate buffer solution at different pH (1.2, 4.0, 6.8, 7.4). The entire system was maintained at 37 °C ± 0.5 °C with gently shaking. Periodically, 0.4 ml of the release medium was withdrawn and the supernatant after separation was stored at 4 °C for UV-vis analysis. The total mass of released 5-fluorouracil (*Q*) at each moment of the experiment was calculated as formula (5), taking into account the aliquots taken. The kinetics of drug release were recorded using a dissolution tester RCZ-1 A coupled with an UV Hewlett Packard spectrophotometer for detection of 5-fluorouracil (5-FU) (λ_max_ = 265 nm) and presented using the diffusion equation as the ratio of the amount of drug released at the time t(M_t_)/ the total amount (M_inf_) of drug released from the tablet.


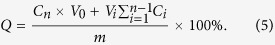


where *Q*, *C*_n_, *C*_i_, *V*_0_ and *m* denote the cumulative release rate (%), the mass concentration of drug release medium after the *n-*th sampling (mg/ml), the mass concentration of drug release after the *i*-th sampling (mg/ml), the volume of the release medium (ml), and the total drug loading (mg), respectively.

## Additional Information

**How to cite this article**: Zheng, X.-F. *et al.* Surface Molecularly Imprinted Polymer of Chitosan Grafted Poly(methyl methacrylate) for 5-Fluorouracil and Controlled Release. *Sci. Rep.* **6**, 21409; doi: 10.1038/srep21409 (2016).

## Figures and Tables

**Table 1:** Selectivity of MIPs and NIPs.

Substrate	Q for MIMs (mg/g)	Q for NIMs (mg/g)	α (imprint factor)
5-fluorouracil(5-FU)	96	14	6.86
Tegafur(TE)	25	11	2.27
uridine(UR)	18	8	2.25
